# Scleromyositis: A distinct novel entity within the systemic sclerosis and autoimmune myositis spectrum. Implications for care and pathogenesis

**DOI:** 10.3389/fimmu.2022.974078

**Published:** 2023-01-26

**Authors:** Margherita Giannini, Benjamin Ellezam, Valérie Leclair, Frédéric Lefebvre, Yves Troyanov, Marie Hudson, Jean-Luc Senécal, Bernard Geny, Océane Landon-Cardinal, Alain Meyer

**Affiliations:** ^1^ Service de Physiologie et explorations fonctionnelles, University Hospital of Strasbourg, Strasbourg, France; ^2^ Centre de Référence des Maladies Autoimmunes Rares, University Hospital of Strasbourg, Strasbourg, France; ^3^ Unité de Recherche 3072 (UR3072), Centre de Recherche en Biomédecine, University of Strasbourg, Strasbourg, France; ^4^ Division of Pathology, Centre Hospitalier Universitaire (CHU) Sainte-Justine, Department of Pathology and Cell Biology, Université de Montréal, Montréal, QC, Canada; ^5^ Division of Rheumatology, Jewish General Hospital, Department of Medicine, McGill University, Montréal, QC, Canada; ^6^ Division of Rheumatology, Centre Hospitalier de l’Université de Montréal (CHUM), Autoimmunity Research Laboratory, CHUM Research Center, Montréal, QC, Canada; ^7^ Department of Medicine, Université de Montréal, Montréal, QC, Canada; ^8^ Division of Rheumatology, Hôpital du Sacré-Coeur, Department of Medicine, Université de Montréal, Montréal, QC, Canada; ^9^ Service de rhumatologie, Centre de Référence des Maladies Autoimmunes Rares, University Hospital of Strasbourg, Strasbourg, France

**Keywords:** myositis, inflammatory myopathies, dermatomyositis, antisynthetase syndrome, systemic sclerosis, scleroderma, scleromyositis, mixed connective tissue disease

## Abstract

Systemic sclerosis and autoimmune myositis are both associated with decreased quality of life and increased mortality. Their prognosis and management largely depend on the disease subgroups. Indeed, systemic sclerosis is a heterogeneous disease, the two predominant forms of the disease being limited and diffuse scleroderma. Autoimmune myositis is also a heterogeneous group of myopathies that classically encompass necrotizing myopathy, antisynthetase syndrome, dermatomyositis and inclusion body myositis. Recent data revealed that an additional disease subset, denominated “scleromyositis”, should be recognized within both the systemic sclerosis and the autoimmune myositis spectrum. We performed an in-depth review of the literature with the aim of better delineating scleromyositis. Our review highlights that this concept is supported by recent clinical, serological and histopathological findings that have important implications for patient management and understanding of the disease pathophysiology. As compared with other subsets of systemic sclerosis and autoimmune myositis, scleromyositis patients can present with a characteristic pattern of muscle involvement (i.e. distribution of muscle weakness) along with multisystemic involvement, and some of these extra-muscular complications are associated with poor prognosis. Several autoantibodies have been specifically associated with scleromyositis, but they are not currently integrated in diagnostic and classification criteria for systemic sclerosis and autoimmune myositis. Finally, striking vasculopathic lesions at muscle biopsy have been shown to be hallmarks of scleromyositis, providing a strong anatomopathological substratum for the concept of scleromyositis. These findings bring new insights into the pathogenesis of scleromyositis and help to diagnose this condition, in patients with subtle SSc features and/or no autoantibodies (i.e. “seronegative” scleromyositis). No guidelines are available for the management of these patients, but recent data are showing the way towards a new therapeutic approach dedicated to these patients.

## 1 Introduction

Systemic sclerosis (SSc) is a rare autoimmune disease characterized by vasculopathy and fibrosis affecting multiple organs (1). Autoimmune myositis (AIM) is another rare condition characterized by myopathy with evidence of inflammation-driven muscle lesions. SSc and AIM are both associated with decreased quality of life (2, 3) and increased mortality (4, 5). However, the prognosis and care largely depend on the subtypes of these diseases, since SSc and AIM both encompass a heterogeneous group of diseases. Identification of these subgroups is fundamental because each requires different management (6). The two predominant forms of SSc are limited cutaneous (lSSc) and diffuse cutaneous scleroderma (SSc) (7). AIM is also a heterogeneous group of myopathies that classically encompasses immune-mediated necrotizing myopathy (IMNM), antisynthetase syndrome (ASS), dermatomyositis (DM) and inclusion body myositis (IBM) (8). The historical entity polymyositis (PM) is now becoming rare and even uncertain, often mistaken for more recently described patterns (6, 9, 10).

Overlap myositis (OM) has been defined as AIM with overlap clinical features (extra muscular involvement other than DM rash) and/or overlap autoantibodies (associated with other connective tissue disease than AIM) (11–13). OM has been shown to be clinically relevant since it has been reported to be the most frequent AIM subgroup and to have diagnostic, prognostic and therapeutic value (11, 12). SSc has been reported to be the most common connective tissue disease in OM patients accounting for about 40% of cases (12, 13). This AIM subgroup associating SSc and OM patients has been denominated “scleromyositis”.

Thus, historically, scleromyositis has been defined as an overlap between SSc and AIM (12, 14, 15). Yet, fulfilling the American College of Rheumatology/European League Against Rheumatism (ACR/EULAR) classification criteria for both SSc (7) and AIM (16) is a definition for scleromyositis (17–19) that is limited by low sensitivity for the condition (20–22).

Whether scleromyositis can be recognized within both the SSc and AIM spectrum has not been reviewed.

Since of these uncertainties, an in-depth review of the literature reporting muscle involvement in SSc was performed, with the objective of better delineating scleromyositis clinically, serologically and histopathologically, and identifying implications of this diagnosis for prognosis and management.

## 2 Methods

### 2.1 An extensive review of the literature was conducted with two research criteria

First, all original articles in English pertaining to SSc where muscle involvement and/or SSc/AIM overlap were directly mentioned or easily calculated from the available data were collected. Second, Pubmed was searched twice in February 2022 and September 2022 using the search words “myositis” or “myopathy” or “myopathies” or “scleromyositis” or “polymyositis” or “dermatomyositis” or “antisynthetase syndrome” or “anti-synthetase syndrome” AND “scleroderma” or “systemic sclerosis” or “scleromyositis” or “anti-PM/Scl” or “anti-PMScl” or “anti-PM Scl” or “PMSCL” or “PM Scl” or “anti-PM-Scl” or “anti-PM75” or “anti-PM100” or “anti-CENPB” or “anti-CENPA” or “anti-CENP-A” or “anti-CENP-B” or “anti-CENPA/B” or “anti-centromere” or “anti–topoisomerase” or “anti-Scl70” or “anti-Scl-70” or “anti-RuvBL1/2” or “anti-RuvBL1” or “anti-RuvBL2” or “anti-ku” or “anti-RNA polymerase III” or “anti-RNA-polymerase III” or “anti-RNA pol” or “anti-POL” or “anti-RNAP III” or “anti-RNPC-3” or “anti-RNPC3” or “anti-RNP” or “anti-U1 RNP” or “anti-U1RNP” or “anti-U3 RNP” or “anti-U3RNP” or “anti-U11/U12 RNP” or “anti-U5 RNP” or “anti-U5RNP” or “anti-SMN” or “anti-fibrillarin” or “anti-Th/To”.

Reference lists of relevant articles were also manually searched to identify additional studies not captured by the search. No restrictions on publication period, type of study, nor setting, were placed on this search. Because there is currently no consensual definition of scleromyositis, all significant descriptions of association between AIM and SSc according to the authors’ opinion were included.

Two reviewers (MG and AM) independently screened titles and abstracts for inclusion. At this stage, animal and pediatric studies were excluded. Records included by at least one reviewer at the title and abstract screening stage were further included for full text review. At this stage, if consensus between the two reviewers was met, each publication was included for data extraction. All studies were included in the data synthesis irrespective of quality assessment.

## 3 Results

Among 3263 references screened, we ultimately included 61 articles published between 1961 and 2022 reporting muscle involvement in SSc and/or SSc/AIM overlap, its characteristics and implication for prognosis and management of patients.

### 3.1 Characteristics of skeletal muscle involvement in systemic sclerosis

According to a 2013 meta-analysis, myositis is reported in 13% (95% CI 10–17) of SSc patients (23).. However, the prevalence ranges widely among surveys, from 5% to 96% (17, 23–48). This important disparity is likely due to the heterogeneity of definitions used for muscle involvement in SSc since no agreement upon diagnostic criteria of muscle involvement in SSc are currently available (49). SSc was diagnosed according to expert opinion (30–36) or ACR 1980 (50) or LeRoy (51) or ACR/EULAR 2013 (7) criteria. Muscle involvement was defined by muscle weakness and/or myalgia and/or amyotrophy and/or muscle enzymes increase and/or myopathic features at electroneuromyography (ENMG) or muscle biopsy and/or oedema at muscle magnetic resonance imaging (MRI) and/or myositis specific/associated autoantibodies or Bohan and Peter criteria (52, 53). When the authors described a myopathy associated with SSc, regardless of the criteria used, we classified these patients as possible scleromyositis. Due to such heterogeneity of definitions for myopathy in SSc, each of the above criteria for muscle involvement has been compared to items included in ACR/EULAR 2017 classification criteria, which are the most up to date criteria for AIM (16).

Conversely, among AIM patients, SSc features are also variably recognized. While the 2017 ACR/EULAR classification criteria for AIM (16) do not recognize OM as a separate entity, up to 29% of AIM cases are reported to have a concurrent SSc diagnosis (12, 18, 54).

Clinical, biological, electromyographic and radiological characteristics of muscle involvement in the whole group of SSc patients and in those possibly suffering from scleromyositis are shown in **Tables 1** and **2**, respectively. Although the prevalence of muscle features varied somewhat according to the composition of the SSc cohorts (relative proportions of lSSc vs dSSc) and/or the criteria selected for the definition of scleromyositis, several characteristic features of skeletal muscle involvement in SSc can be drawn and are discussed herein.

**Table 1:** Prevalence of muscle features in systemic sclerosis patients.

First author, year of publication	SSc criteria	N patients (lSSc/dSSc)	Myalgia (%)	Weakness				Muscle enzymes		ENMG		Muscle MRI (oedema/fatty infiltration/fasciitis) (%)
				Patient-reported (%)	Proximal at examination (%)	Distal at examination (%)	Muscle atrophy (%)	↑CK (%)	↑ aldolase (%)	Myopathic (%)	Neuropathic (%)	
Medsger, 1968	Study specific	53	11	>60	53	68	–	9	57	–	–	–
Thompson, 1969	Study specific	15	–	–	20	–	20	–	–	27	–	–
Clements, 1978	Study specific	24 (2/22)	–	–	83	–	–	46	75	92	–	–
Hausmanowa-Petrusewicz, 1982	Study specific	39 (21/18)	–	–	–	–	–	–	–	74 proximal; 49 distal	–	–
West, 1981	Study specific	47 (16/31)	–	–	>6	–	–	>17	>15	>11	–	–
Russell, 1983	ACR 1980	28	–	–	32	–	–	29		61	–	–
Averbuch-Heller, 1992	Study specific	50	–	–	22		–	22		22	4	–
Follansbee, 1993	ACR 1980	1095	–	–	–	–	–	19	–	–	–	–
Hietaharju, 1993	ACR 1980	32 (23/9)	22	–	25	–	9	21	–	22	22	–
Clements, 1999	ACR 1980	134 (0/134)	35	33	11	–	–	8	–	–	–	–
Mimura, 2005	ACR 1980	302 (169/133)	–	–	≥14	–	–	–	–	11	–	–
Walker 2007	ACR 1980	3656 (2101/1349)	–	29		–	15	7	–	–	–	–
Meier, 2012	ACR 1980	7655 (4481/2838)	–	25		–	12	9	–	–	–	–
Partovi, 2012	ACR 1980	11 (5/3)	–	–	9	9	9	18	–	–	–	–
Tolédano, 2012	ACR 1980	137	56	–	10	–	–	–	37	–	–	–
Schanz, 2013	ACR 1980 and MS	18 (3/15)	17	28	–	–	–	47	–	–	–	78/–/89
Bhansing, 2014	ACR 1980, LeRoy 2001	385 (276/109)	–	5		–	–	12	–	–	–	–
Jung, 2014	ACR 1980	947 (566/381)	–	–	–	–	–	6	–	–	–	–
Paik, 2016	ACR 1980, study specific	1718 (1034/684)	–	–	23	–	–	–	–	–	–	–
Corallo, 2017	ACR/EULAR 2013	112	–	–	31	–	–	18	11	31	–	–
Zhou, 2020	ACR 1980, ACR/EULAR 2013	204 (122/82)	≥12		–	–	–	≥22	–	–	–	–
Siegert, 2021	ACR/EULAR 2013	367 (231/136)	–	–	50	–	–	–	–	–	–	–
Ross, 2022	ACR/EULAR 2013	32 (13/19)	–	–	13	–	–	19	–	–	–	41/16/–

ACR, American College of Rheumatology; MS, musculoskeletal involvement; SSc, systemic sclerosis.

**Table 2:** Prevalence and characteristics of scleromyositis in systemic sclerosis patients.

Author, year of publication	SSc criteria	N patients (lSSc/dSSc /sine scleroderma)	Myositis criteria			MUSCLE FEATURES % of scleromyositis									
				Sclero myositis/SSc (%)	Myalgia (%)	Weakness					Muscle enzymes		ENMG		Muscle MRI (oedema/fatty infiltration/fascitis) (%)
						Patient-reported (%)	Proximal at examination (%)	Distal at examination (%)	Axial at examination (%)	Muscle atrophy (%)	↑CK (%)	↑ aldolase (%)	Myopathic (%)	Neuropathic (%)	
Tuffanelli, 1961	Study specific	727 (688/39/0)	Study specific	5	–	–	–	–	–	–	–	–	–	–	–
Medsger, 1968	Study specific	16	Severe to marked weakness	30	38	–	100	–	–	–	–	82	–	–	–
Clements, 1978	Study specific	23 (2/21/0)	Weakness or ↑CK/aldolase or myopathic ENMG	96	–	–	87	–	–	–	48	74	96	–	–
West 1981	Study specific	8 (0/8/0)	↑CK(>196 mµ/ml) or aldolase(>10U/l)+ ≥1 among: proximal weakness, myopathic ENMG or muscle biopsy	17	–	–	>63	–	–	–	100	88	63	–	–
Hausmanowa-Petrusewicz, 1982	Study specific	29 (15/14/0)	myopathic ENMG	74	–	–	–	–	–	–	–	–	74	–	–
Russell, 1983	ACR 1980	9	Quadriceps weakness	32	–	–	100	–	–	–	33		86		–
Mimori, 1987	ACR 1980	27	Bohan and Peter	8	88	100	–	–	–	–	96	–	92		–
Ringel et al.,1990	LeRoy 1985	14 (0/14/0)	Symmetrical proximal weakness	–	21	100	100	–	–	–	67	–	100	21	–
Averbuch-Heller, 1992	Study specific	11	Weakness + CK or aldolase >1ULN + myopathic ENMG or muscle biopsy	22	–	–	100	–	–	–	100		100	18	–
Follansbee, 1993	ACR 1980	25 (3/18/–)	Weakness + CK>1ULN or myopathic ENMG or muscle biopsy	17	–	–	64	–	–	–	84	64	53	–	–
Hietaharju, 1993	ACR 1980	5 (3/2/0)	≥3 among: symmetrical weakness (limb-girdle and anterior neck), ↑CK ≥220 U/L, myopathic ENMG or muscle biopsy	16	–	–	100	–	–	80	60	–	100	20	–
Mimura, 2005	ACR 1980	43 (16/27/0)	Weakness + CK<1ULN or myopathic ENMG or muscle biopsy	14	–	–	100	–	–	–	–	–	76	–	–
Ranque,2009	ACR 1980, LeRoy 2001	35 (9/26/0)	Weakness and myalgia and/or CK>5ULN + myopathic ENMG or muscle biopsy	–	86	–	77	–	–	–	82	76	93	29	67/25/–
Ranque 2010	ACR 1980, LeRoy 2001	40 (10/30/0)	Weakness and myalgia and/or CK>5ULN + myopathic ENMG or muscle biopsy	–	83	–	78	–	–	–	82	80	94	18	77/23/–
Tolédano 2012	ACR 1980	9 (4/5/0)	Weakness + ≥ 2 among: muscle oedema on thigh MRI, myopathic ENMG or muscle biopsy	7	–	–	100	–	–	–	67	100	89	–	78/–/–
Schanz 2013	ACR 1980	14 (0/14/0)	Muscle oedema and hyperaemia on whole-body MRI	78	–	–	–	–	–	–	50	–	–	–	100/–/93
Bhansing, 2014	ACR 1980, LeRoy 2001	25 (19/6/0)	Bohan and Peter	6	NA	100	–	–	–	–	96	–	88	–	–
Jung, 2014	ACR 1980	53 (32/21/0)	CK ≥200 µ/L (women) or ≥250 µ/L (men)	6	–	–	13	–	–	–	100	–	–	–	–
Paik, 2015	ACR 1980, study specific	42 (15/27/0)	Weakness that lead to muscle biopsy	–	–	–	100	–	–	–	74	–	90	44	88/29/–
Corallo, 2017	ACR/EULAR 2013	35 (11/24/0)	Weakness, ↑CK/aldolase, myogenic ENMG	31	–	–	100	–	–	–	57	34	100	–	–
De Lorenzo, 2018	ACR/EULAR 2013	41	Anti-PM/Scl antibodies	–	68	–	93	23	–	Deltoid^$^	–	–	40	–	39/50/56
Zhou 2020	ACR 1980, ACR/EULAR 2013	204 (122/82/–)	Weakness/fatigue/muscle pain + ≥ 1 feature among: CK>145U/l, myopathic ENMG or muscle biopsy or oedema/atrophy/hyperemia at MRI	22	≥55		–	–	–	–	100	–	≥27	–	≥9
Baumberger, 2021	ACR/EULAR 2013	58 (37/18/3)	≥ 1 feature among: proximal weakness, amyotrophy, ↑CK/aldolase, myositis-specific/associated Abs	13	–	–	40	–	–	14	52	–	–	–	38
Ellezam 2021	ACR/EULAR 2013	33 (8/12/13)	≥2 experts opinion*	–	–	–	88	–	–	–	–	–	93	–	–
Siegert 2021	ACR/EULAR 2013	18 (13/5/0)	Proximal weakness and myopathic muscle biopsy	5	–	–	100	–	–	–	60	–	–	–	–
Ross 2022	ACR/EULAR 2013	13 (1/12/0)	Muscle oedema at MRI	41	–	–	15	–	–	–	8	–	–	–	100/31/–
Ellezam 2022	ACR/EULAR 2013, expert opinion*	60 (20/16/24)	Weakness + CK>200 IU/l and/or myopathic ENMG	–	–	–	77	34	21	–	–	–	88	–	–
Leclair 2022	ACR/EULAR 2013, expert opinion*	42 (15/14/13)	Any of muscle weakness, CK elevation, myopathic EMG or muscle biopsy	–	–	–	86	49	29	–	83	–	91	–	82
Matas-García 2022	ACR/EULAR 2013, LeRoy 2001	40 (–/24/–)	Proximal weakness + CK>2ULN and/or myopathic ENMG	–	60	–	92	–	35	18	63	74	72	25	–

Abs, antibodies; CK, creatine kinase; ENMG, electroneuromyogram; MRI, magnetic resonance imaging; ULN, upper limit of normal. *expert opinion: consensus of ≥2 experts. ^$^: the frequency has not been reported.

#### 3.1.1 Clinical characteristics of muscle involvement

Myalgias are reported by 11% to 56% of SSc patients (30, 38, 45, 46, 55, 56) compared to 21 to 88% of patients with scleromyositis (27, 28, 30, 41, 57–59). Thus, in SSc, as in other settings (16, 60, 61), myalgia lacks sensitivity and specificity for the diagnosis of myopathy. Myalgia is not included in the 2017 ACR/EULAR criteria for AIM and was selected as a criterion for scleromyositis only by a single group (27, 28).

Subjective muscle weakness was reported by 23% to 60% of SSc patients (30, 44, 46, 55) while objective muscle weakness was detected on physical examination in 9 to 83% of cases (24, 25, 30–32, 37, 38, 40, 42, 45, 47, 55, 56, 62). This may indicate that extra-muscular involvement (i.e. joint, skin thickening, interstitial lung disease [ILD], anemia and pulmonary arterial hypertension [PAH]) also contributes to the self-reported exercise limitation in some SSc patients. Muscle weakness on physical examination is a 2017 ACR/EULAR criterion for AIM whereas self-reported weakness is not (16). It was selected as a criterion for scleromyositis by most of the studies. Depending on the definition used, muscle weakness was reported in 13% to 100% of scleromyositis patients (17, 20, 21, 24–30, 32, 34, 35, 37, 38, 41–43, 45, 47, 57–59, 63, 64).

The distribution of muscle weakness was generally symmetrical and proximal both in the upper and lower limbs. Weaker upper limbs than lower limbs has been reported to be a feature of scleromyositis as compared to other myositis subgroups (57). More rarely, scleromyositis (23-49%) and SSc patients (9-68%) presented with distal weakness (20, 21, 30, 57, 62). However, skin thickening, joint contractions and arthritis may also contribute to distal muscles weakness. Finally, axial involvement leading to head drop syndrome and/or camptocormia has been reported up to one-third of scleromyositis patients (20, 21, 59, 65–68).

Muscle weakness, both self-reported and at examination, was more common in dSSc compared to lSSc (40, 44). The frequency of muscle atrophy was also greater in dSSc as compared to lSSc (40, 44), indicating a more damaging myopathy in this subgroup. Muscle weakness has been independently associated with different degrees of physical disability (56).

#### 3.1.2 Muscle enzymes

Serum CK level elevation is a 2017 ACR/EULAR criterion for AIM (16) and it is reported in 6% to 47% of SSc patients (17, 24, 26, 29, 30, 32, 38, 40, 44, 46, 47, 55, 62). Serum CK level elevation was selected as a criterion for scleromyositis in about half of the studies reviewed and was noted in 8% to 100% of scleromyositis patients (17, 24–29, 32, 34, 37, 41, 45–47, 58, 59, 64). Among the patients of the Canadian Scleroderma Research Group cohort, a worse survival was recorded in patients with elevated serum CK levels (29). However, in scleromyositis patients, serum CK levels > 5 times the upper limit of normal (ULN) were associated with better myositis response to corticosteroid treatment, as compared to lower serum CK levels (27). It should be noted that CK levels are also elevated in myocarditis, an important diagnostic consideration in SSc (69). Measurement of the isoenzymes (MM and MB) and serum troponins (t and i) levels are notably useful to distinguish whether the increased CK level is of skeletal and/or cardiac muscle origin (70).

Serum aldolase levels are more frequently increased than CK levels in SSc patients (11 to 75% of patients) (24, 30, 32, 45). Elevated serum level of aldolase is also a 2017 ACR/EULAR criterion for AIM (16). In a prospective cohort of SSc patients without weakness at baseline, elevated serum aldolase levels had a higher predictive value for detection of incident myopathy than elevated CK levels (45). However, unlike CK, aldolase activity is present in many tissues (71) and the specific tissue source of the increased aldolase in SSc patients remains to be determined. It was selected as a criterion for scleromyositis in only two studies (24, 37), and was increased in 34 to 100% of scleromyositis patients (21, 24, 26–28, 30, 32, 34, 45, 59).

#### 3.1.3 Electroneuromyography

ENMG was abnormal in 11 to 92% of SSc patients (24, 31–33, 38, 42). Although ENMG is generally very useful to explore the cause of weakness and/or elevated serum levels of muscle enzymes, it was not retained as a 2017 ACR/EULAR criterion for AIM (16). It was selected as a criterion for scleromyositis by about half of the studies reviewed and a myopathic pattern was found in 40 to 100% of scleromyositis patients (17, 20, 21, 24, 26–28, 32, 41, 42, 45, 57–59, 63, 64). Consistent with the reported clinical distribution of weakness, ENMG more frequently demonstrated myopathic patterns in proximal than in distal muscles (33). Among those myopathic patterns, spontaneous activity (positive sharp waves and/or fibrillation) has been associated with the highest CK levels (72). Interestingly, neuropathic pattern has been also reported in 18 to 29% of scleromyositis patients (27, 28, 59).

#### 3.1.4 Muscle magnetic resonance imaging

MRI allows non-invasive visualization of characteristic myositis changes, including edema, fatty replacement, atrophy, fasciitis, and subcutaneous pathology (73). However, none of these features are entirely specific to AIM (74) and therefore can lead to misdiagnosis when MRI findings are not interpreted according to the clinical context (75). Thus, muscle MRI findings were not selected as a 2017 ACR/EULAR criterion for AIM (16).

Abnormal muscle findings on whole body MRI have been found in 41 to 89% of SSc patients with musculoskeletal symptoms (muscle weakness, myalgia, arthralgia, tendon sheath discomfort and/or tendon friction rub) (46, 47). It was chosen as a criterion for scleromyositis in only three studies and 38% to 100% of scleromyositis patients had abnormal MRI muscle findings (27, 28, 37, 45, 47, 57, 64). In scleromyositis patients, MRI myositis lesions include fasciitis (thickening and/or increased signal intensity of the fascia on STIR and post-gadolinium images) and/or perifascial edema and/or muscle edema (27, 28, 46). These findings resemble those observed in DM (76). Fascial and/or muscle lesions were correlated with muscle weakness, Rodnan’s score and C-reactive protein levels (but not with serum CK levels) (46).

#### 3.1.5 Muscle biopsy

Myopathological analysis is an important tool to help differentiate AIM from muscular dystrophies and other non-inflammatory myopathies and to identify AIM subgroups. Myopathological lesions in AIM encompass several tissue domains that include muscle fibers, connective tissue and vasculature (77). These lesions define histopathological patterns, some of which have been linked with clinical features and outcomes. During the 119th European NeuroMuscular Centre (ENMC) international workshop, experts reached a consensus that histopathological patterns in AIM include PM, DM, IMNM, IBM and non-specific myositis (78). Thus, there is currently no estabilished histopathological criteria for scleromyositis and this entity is not recognized by the ENMC as a distinct AIM subset.

Myopathological lesions in scleromyositis patients are summarized in **Table 3**.

**Table 3:** Muscle histopathological features of scleromyositis.

MYOPATHOLOGICAL LESIONS	N biopsies featuring the lesion/N biopsies (%)	Range %	N studies reporting the lesion
**Number of biopsies**	637		58
**Number of necropsies**	21		2
**INFLAMMATORY INFILTRATE**			
**Inflammatory infiltrates**	294/516 (57%)	0-100%	50
**Perimysial inflammatory infiltrates**	45/92 (49%)	0-100%	12
**Endomysial inflammatory infiltrates**	73/143 (51%)	14-100%	18
**Perivascular inflammatory infiltrates**	116/216 (54%)	0-100%	21
**Invasion of non-necrotic fibers**	11/45 (24%)	0-80%	10
**CD3+ cells**	77/157 (49%)	18-100%	15
**CD4+ cells**	16/50 (32%)	15-100%	4
**CD8+ cells**	10/45 (22%)	18-18%	4
**CD20+ cells**	25/72 (35%)	15-61%	7
**MUSCLE FIBER DOMAIN**			
**Atrophy**	173/294 (59%)	14-100%	25
**Perifascicular atrophy**	6/31 (19%)	1-33%	6
**Regenerative characteristics**	87/195 (45%)	0-83%	17
**Necrosis**	219/388 (56%)	17-100%	30
**MHC-I expression**	114/147 (78%)	56-100%	16
**MHC-I diffuse**	3/56 (11%)	N/A	5
**MHC-I perifascicular**	30/54 (56%)	N/A	4
**MHC-II expression**	33/60 (55%)	0-57	2
**C5b-9 deposition (unspecified)**	28/72 (39%)	0-50%	7
**Sarcolemmal C5b-9 deposition**	33/70 (47%)	45-50%	4
**Neurogenic changes**	51/231 (22%)	0-55%	12
**Mitochondrial abnormalities**	9/117 (8%)	0-30%	9
**Rimmed vacuoles**	11/34 (32%)	0-29%	11
**CONNECTIVE TISSUE DOMAIN**			
**Fibrosis**	205/456 (45%)	17-100%	22
**VASCULAR DOMAIN**			
**Vasculopathy**	121/207 (58%)	19-100%	10
**Basement membrane abnormalities**	67/100 (67%)	19-75%	5
**Capillary C5b-9 deposition**	37/128 (26%)	0-50%	6
			

MHC, major histocompatibility complex; n, number; N/A, not applicable.

In a recent scoping review (79), the most frequent myopathological lesions included sarcolemmal expression of class I major histocompatibility complex (72%), myofiber necrosis (56%), inflammatory infiltrates (57%), endomysial fibrosis (33%) and vasculopathy (33%). When these basic lesions were integrated according to the ENMC consensus, IMNM and PM were the two dominant patterns, present in up to 60% and 78% of scleromyositis patients, respectively.

Yet, several recent studies indicate that vasculopathy and fibrosis might be pathological hallmarks of scleromyositis that distinguish them from other AIM sugroups. Indeed, these two features recapitulate two cardinal pathophysiological processes of SSc (80, 81) supporting the concept of SM as an organ manifestation of SSc and a distinct subset of AIM (20).

**Figure 1:**
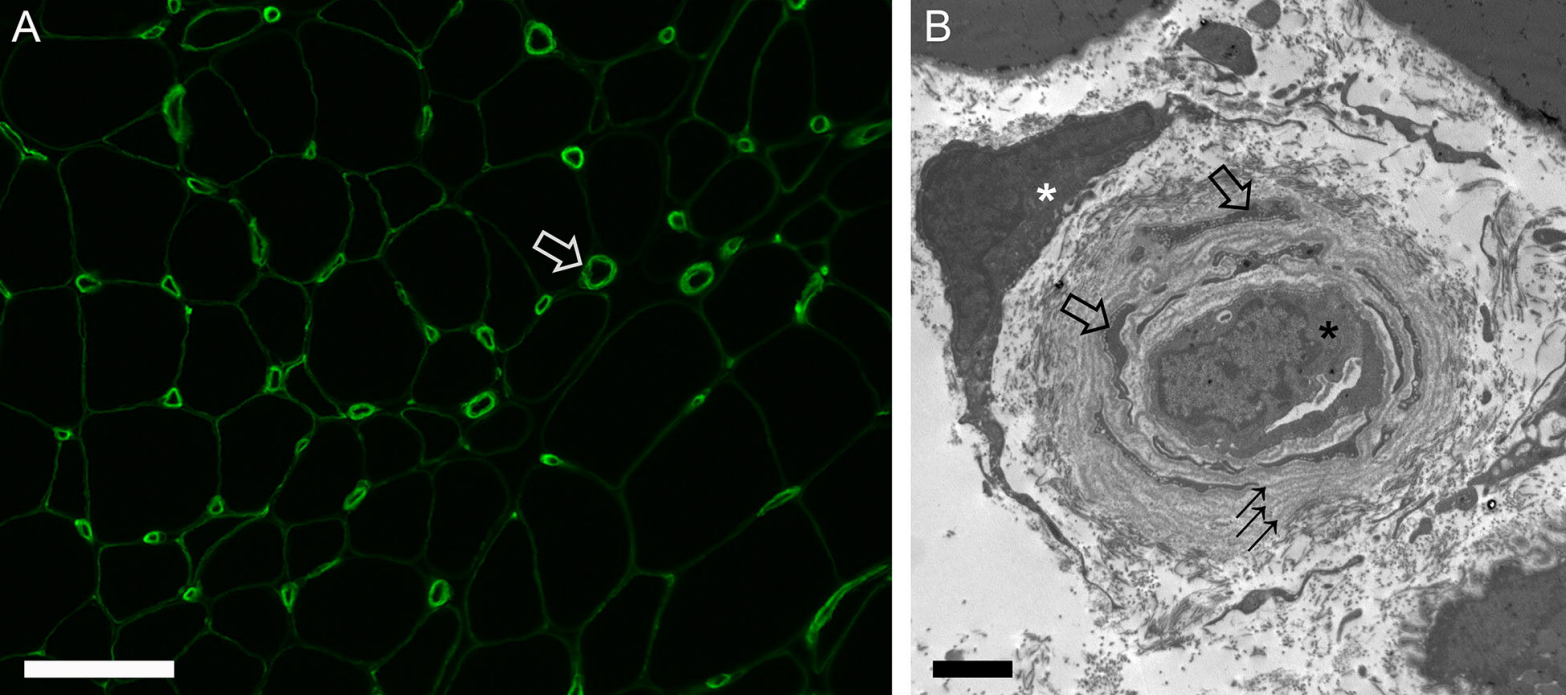
Capillary pathology in scleromyositis. **(A)** Immunofluorescence for collagen type 4 highlighting enlarged endomysial capillaries often with laminated appearance of basement membrane (open arrow). **(B)** Electron micrograph showing a collapsed capillary (black asterisk) with several concentric layers of reduplicated basement membrane (arrows) ensheathing many pericyte processes (open arrows). A fibroblast is also shown (white asterisk). Bars: A, 100 µm, B, 2 µm.

Vasculopathy in scleromyositis has been shown in the form of capillary dropout and enlargement on light microscopy, abnormal capillary expression of VEGF or PDGFR-β on immunohistochemistry, and basement membrane reduplication with endothelial activation and/or increased numbers of ensheathed pericyte processes on electron microscopy (20, 24, 25, 63). In a recent study, capillary pathology with prominent basement membrane reduplication (≥4 layers in >50% of capillaries) was found in 65% of scleromyositis vs 0% of other AIM controls (p<0.001) (20) (**Figure 1**). This hallmark histopathological feature of scleromyositis provides a strong anatomopathological substratum for the thus far clinically-derived concept of scleromyositis Moreover, a predominant fibrotic pattern without inflammation or necrosis (“fibrosing myopathy”) has been described in 20-27% of scleromyositis cases and was associated with poor response to immunomodulatory drugs and increased risk of death from cardiac involvement (59, 72). On the other hand, necrosis and inflammation have been associated with good responses to those drugs (27, 59). Interestingly, “fibrosing myopathy” has generally been associated with lower increases in CK level and lack of spontaneous activity (positive sharp waves and/or fibrillation) on ENMG, while the opposite was found in scleromyositis patients with muscle inflammation and/or necrosis (27, 59, 72).

### 3.2 Extra-muscular involvements

As compared to SSc without muscle involvement and to the other AIM subgroups, scleromyositis patients present with distinct extra-muscular complications, some of which are associated with poor prognosis.

Scleromyositis patients have been shown to have a worse survival rate than SSc patients without myositis, the most common cause of death being cardiopulmonary disease (42% to 63%) (29, 82). On the other hand, among scleromyositis patients, SSc-related complications accounted for up to half of the deaths (12). This indicates that scleromyositis is not merely an overlap between SSc and AIM, but a unique condition within both the SSc and AIM spectrum.

#### 3.2.1 Lung involvement

In the EUSTAR cohort, more than 50% of SSc-related deaths were of pulmonary causes (83). A recent meta-analysis showed that the prevalence of SSc-ILD was 56% (95% CI 49%- 63%), with high heterogeneity across studies (84). Clinically significant ILD is present in approximately 40% of patients with SSc (85), and high resolution computed tomography (HRCT) can detect interstitial abnormalities in up to 90% of the patients (86). Lung involvement negatively impacts survival (29) and quality of life (87).

Several studies have shown that scleromyositis patients more frequently present with ILD than SSc patients without muscle involvement (up to 68% vs 13%) (27–29, 42, 56, 82).

Although involvement of respiratory muscles in SSc as well as in scleromyositis has not been systematically examined, it has been described in case reports and series (21, 88–90). Moreover, in the EUSTAR cohort, patients with ILD and anti-PM/Scl (an autoantibody that is associated with scleromyositis as detailed below), vital capacity tended to improve whereas diffusion capacity of lung for carbon monoxide remained stable at one-year follow-up (91). This suggests that respiratory muscle weakness contributes to respiratory impairment in this subgroup.

#### 3.2.2 Heart involvement

In the EUSTAR cohort, 26% of SSc-related deaths were related to cardiac involvement, being the second cause of mortality in SSc patients (83). The clinical presentation may include arrhythmias (92%), congestive heart failure (68%), conduction system abnormalities (60%) and, rarely, pericardial effusion, although most patients are asymptomatic in the early stages (26, 92). Echocardiography demonstrate impaired left and right ventricle systolic dysfunction in approximately 20% of SSc patients (93). Conversely, cardiac magnetic resonance (CMR) abnormalities, such as T2-weighted changes indicating myocardial edema and late gadolinium enhancement measuring focal fibrosis, are detected in up to 96% of SSc patients even in early phases of the disease and without significant impairment of cardiac function (93–99).

Myopathy is an independent risk factor for cardiac involvement in SSc patients (26, 28, 34, 42, 100–102). Myocardial disease has been reported in up to 21% of scleromyositis patients versus 10% of SSc patients (26), although screening tools and definitions used for cardiac involvement were heterogeneous.

It should be noted that an elevation of serum CK levels can reflect either skeletal or cardiac muscle disease. Among SSc patients, those with abnormal CK levels more frequently fulfilled criteria for myocardial disease (23% vs. 9%) (e.g. atrial or ventricular arrhythmias requiring therapy, or congestive heart failure), and died of cardiac causes (9% vs 3%) (26) than those with normal CK levels. Thus, prompt recognition of heart involvement, taking advantage of CMR and cardiac biomarkers specific to the myocardium, such as cardiac troponin I (70), is recommended to adapt treatment strategy.

#### 3.2.3 Scleroderma renal crisis

Scleroderma renal crisis (SRC) is the third life-threatening complication, accounting for 8% of SSc-related deaths (83). While SRC is reported in about 5% of the SSc patients (103), it occurs in up to 15% of the scleromyositis patients (27, 28, 56, 64).

In a cohort of 1718 SSc patients, those with muscle weakness more frequently developed SRC than patients without (7.1% vs 1.4%) (56). Corticosteroids (CS) are frequently used in scleromyositis (104) and are associated with SRC (103). In the EUSTAR cohort, while the risk of SRC was significantly increased in anti-PM/Scl positive patients as compared to the rest of SSc patients, multivariate analysis revealed that SRC was associated with CSbut not with anti-PM/Scl antibodies (91). This may indicate that CS use rather than anti-PM/Scl antibody itself increases the risk of SRC in SSc patients.

#### 3.2.4 Gastro-intestinal involvement

Gastrointestinal complications of SSc decrease the quality of life (87, 105) and cause death in about 4% of patients (83, 106).

Esophageal involvement manifests as dysphagia, acid reflux, heartburn and retrosternal pain (107). Long-term esophageal motility disorders in patients with SSc may lead to complications, such as gastroesophageal reflux, esophagitis, esophageal erosion, stricture, ulcers, diverticulum, leukoplakia, Barrett’s esophagus and adenocarcinoma (107). Although the causal relationship between gastroesophageal reflux and ILD remains unclear, early treatment of esophageal disease could reduce the severity of lung involvement (108). Manifestations related to small and large intestine dysfunctions and anorectal impairment include postprandial bloating, abdominal distension and pain, constipation and fecal incontinence, diarrhea, at times explosive, malabsorption, which can lead to severe malnutrition (109).

The frequency of these complications is similar in SSc patients with and without muscle involvement (approximately 65%) (41, 42, 44, 110), However, gastrointestinal manifestations are frequently more severe in scleromyositis patients as compared to patients without myositis (56).

#### 3.2.5 Joint and tendon involvements

Tendon friction rubs and synovitis are linked with decreased quality of life (87, 111) and disease progression in SSc (112).

Both complications are more frequently reported in scleromyositis than in SSc patients without muscle involvement (29, 56). Moreover, muscle fascial enhancement on MRI has been linked with synovitis (46).

#### 3.2.6 Skin involvement

Skin involvement negatively impacts quality of life (87), due to skin tightening, pain, pruritis and functional limitation, mainly hand disability (113). It also contributes to functional disability with positive correlation between skin severity and Health Assessment Questionnaire scores (114, 115). Moreover, survival is reduced in patients with high skin involvement score (116–118). Some evidence suggests that skin changes indicate changes in visceral disease severity (116, 117, 119). In accordance with this view, except for those with distinct autoantibody profiles as discussed below, scleromyositis patients generally have more diffuse than limited skin thickening (40-75% vs 25-60%, respectively) (27–29, 42, 44, 56, 64) and have higher modified Rodnan skin score (mRSS) compared to SSc (29, 56).

### 3.3 Autoantibodies

None of the 3 SSc-specific autoantibodies included in the 2013 ACR/EULAR criteria for SSc (anti-centromere, anti–topoisomerase I [anti–Scl-70], anti–RNA polymerase III) (7) have been strongly associated with scleromyositis. Indeed, anti-centromere antibodies have been consistently reported to be negatively associated with muscle involvement in SSc (18, 26, 28, 120). Anti-topoisomerase autoantibodies, when present (0% to 69% of scleromyositis patients) (17, 18, 24, 25, 27–29, 41, 42, 45–47, 56, 64, 72, 82, 121), have been associated with fibrosis on muscle biopsy (72), in accordance with the diffuse skin thickening phenotype of these patients.

In contrast, several other autoantibodies (not included in the SSc 2013 ACR/EULAR criteria), such as anti-PM/Scl, -Ku, -U1-RNP, -U3-RNP, - RuvBL1/2, -SMN, have been specifically associated with scleromyositis. This contributes to the low sensitivity of ACR/EULAR criteria to identify scleromyositis (22), and also indicates that scleromyositis may be driven by different autoimmune mechanisms among the SSc spectrum. Autoantibody expression varies widely among patients and each of the scleromyositis autoantibodies have been associated with specific clinical phenotype, response to treatment and prognosis, as summarized in **Table 4**.

**Table 4:** Syndromes delineated by autoantibodies associated with scleromyositis.

					ORGAN INVOLVEMENTS FREQUENCY												
Author	Antibody Anti	Case/Control, n/N	Age at SSc onset	Male sex	Muscle	Joint	ILD	PAH	Heart	SRC	Diffuse skin	DM rash	Calcinosis	Gastro-intestinal	Digital ulcers	Teleangiectasia	Cancer
Reimer, 1988	-PM/Scl	8/128	↓ (vs no ANoA)	=	↑ (vs no ANoA, trend)	↑	=	NA	↓ (trend)	↑ (trend)	=	NA	↑ (vs. U3 RNP, trend)	↓ (trend)	NA	=	NA
Steen, 2005	-PM/Scl	36/927	↓	=	↑	=	= (↑severity)	=	=	=	↓	NA	↑	= (↓severity)	=	NA	NA
Hanke, 2009	-PM/Scl	35/245	trend to ↓	NA	↑	NA	↑	=	=	=	=	NA	NA	↓	↑	NA	NA
Mierau, 2011	-PM/Scl	42/863	=	=	↑	=	=	=	=	=	=	–	–	↓	–	–	–
Graf, 2012	-PM/Scl	9/120	NA	=	NA	NA	=	=	NA	NA	↓ (trend)	NA	NA	NA	NA	NA	NA
Koschik, 2012	-PM/Scl	76/2349	↓	NA	↑	=	↑	↓	=	=	↓	NA	↑	↓	↓	NA	NA
D’Aoust, 2014	-PM/Scl	26/708	↓	NA	↑	↑	=	=	=	=	↓ (trend)	NA	↑	↓ (↓severity)	=	NA	NA
Guillen-Del Castillo, 2014	-PM/Scl	14/49	=	NA	↑	=	=	=	=	=	↓	NA	=	↓	↑	NA	NA
Kaji, 2014	-PM/Scl	76/37 (RuvBL1/2)	↓	↓	=	NA	=	=	↓ (trend)	=	↓	↑	NA	↓	=	NA	NA
Kaji, 2014	-PM/Scl	76/44 (Ku)	=	=	=	NA	=	↓ (trend)	=	=	=	↑	NA	trend to ↓	=	NA	NA
Lazzaroni, 2021	-PM/Scl	144/7058	=	↑	↑	=	↑	=	↓	↑ (trend)	=	↑	↑	↓	↓	=	=
Kuwana, 1994	-Ku	7/267	↓	↓	↑	=	=	=	=	=	NA	NA	NA	=	↓	NA	NA
Rozman, 2007	-Ku	14/611	matched	matched	↑	↑	=	↑	=	=	↓	NA	=	NA	↓	↓	NA
Cavazzana, 2008	-Ku	8/371	↑	↓	↑	NA	↑	NA	NA	NA	NA	NA	NA	NA	NA	NA	NA
Mierau, 2011	-Ku	10/863	=	=	↑	=	=	=	=	=	=	–	–	=	–	–	–
Rodriguez-Reyna, 2011	-Ku	14/125	NA	NA	=	=	=	↑	↑	=	=	NA	NA	=	↓	NA	NA
Graf, 2012	-Ku	6/123	NA	=	↑	NA	=	NA	NA	NA	↓ (trend)	NA	NA	NA	NA	NA	NA
Cavazzana, 2013	-Ku	13/67(CENPB)	NA	↑	↑	↑	↑	NA	NA	NA	=	NA	NA	=	↓	NA	NA
Kaji, 2014	-Ku	44/37 (RuvBL1/2)	↓	↓ (trend)	=	NA	=	=	=	=	↓	=	NA	=	=	NA	NA
Patterson, 2015	-Ku	14/491	NA	NA	NA	NA	NA	NA	NA	NA	NA	NA	NA	NA	NA	↓	NA
Hoa, 2016	-Ku	24/2116	↑	↓	↑	=	↑	↑	NA	=	↓	NA	↓	↑	↓	=	=
Kuwana, 1994	-U1 RNP	67/179	↓	=	=	↑	=	↑	=	=	=	NA	NA	=	=	NA	NA
Jacobsen, 1998	-U1 RNP	15/215	↓	=	↑	↑	=	NA	=	=	↓	NA	=	=	=	=	=
Ihn, 1999	-U1 RNP	18/205	=	=	NA	↑	↑	NA	=	=	=	NA	=	=	=	NA	=
Asano, 2003	-U1 RNP	17/11	↑	=	=	=	↑	NA	=	↓ (trend)	=	NA	↓ (trend)	=	=	=	NA
Steen, 2005	-U1 RNP	71/892	↓	=	↑	↑	= (↑severity)	=	=	=	↓	NA	=	= (↑severity)	=	NA	NA
Mierau, 2011	-U1 RNP	39/863	↓	=	↑	↑	=	=	=	=	=	–	–	=	–	–	–
Graf, 2012	-U1 RNP	9/120	NA	=	↑ (trend)	NA	=	↑	NA	NA	NA	NA	NA	NA	NA	NA	NA
Reimer, 1988	-U3 RNP	22/114	↓ (vs no ANoA)	↑ (trend)	=	↓ (vs no ANoA)	=	NA	↑ (trend)	=	=	NA	↓ (trend)	=	NA	=	NA
Okano, 1992	-U3 RNP	24/392	↓	NA	↑	=	=	↑	=	=	=	NA	↑	↑	↑	=	NA
Kuwana, 1994	-U3 RNP	10/265	=	=	=	↓	↓	=	=	=	↑ (trend)	NA	NA	=	=	NA	NA
Arnett, 1996	-U3 RNP	27/267	NA	↑	↑	NA	↑	=	↑	↑	↑ (vs anti-CENPB)	NA	NA	↑	NA	NA	NA
Jacobsen, 1998	-U3 RNP	8/222	=	=	=	=	=	NA	=	=	=	NA	=	=	=	=	NA
Falkner, 2000	-U3 RNP	24/268	NA	NA	↑ (trend)	↑ (vs anti-CENPB)	↓	↑	↑	↓	=	NA	NA	↑	NA	NA	NA
Yang, 2003	-U3 RNP	31/161	=	=	=	↑	↑	NA	=	=	↑	NA	NA	↑	NA	NA	NA
Steen, 2005	-U3 RNP	55/908	↓	=	↑	↑	= (↑severity)	↑	= (↑severity)	↑	↑	NA	↑	= (↑severity)	=	NA	NA
Aggarwal, 2009	-U3 RNP	108/2471	↓	↑	↑	=	=	↑	=	=	↓	NA	NA	=	NA	NA	NA
Mierau, 2011	-U3 RNP	12/863	↓	=	↑	=	=	=	=	=	↑	–	–	=	–	–	–
Kaji, 2014	-RuvBL1/2	10/578	↑	↑	↑	NA	=	=	↑	=	↑	NA	NA	=	=	NA	NA
Kaji, 2014	-RuvBL1/2	27/458	=	=	↑	NA	=	=	=	=	↑	NA	NA	↑ (trend)	↓	NA	↑ (trend)
Landon-Cardinal, 2020	-SMN	5/81	NA	↓ (trend)	↑ (trend)	=	↓ (trend)	NA	NA	↓ (trend)	=	NA	↑ (trend)	=	=	=	=

ANoA, antinucleolar antibodies; DM, dermatomyositis; ILD, interstitial lung disease; NA, not assessed; PAH, pulmonary arterial hypertension; SRC, scleroderma renal crisis; ↑, increased (p<0.05); ↓, decreased (p<0.05). = equal. Trend (p>0.05): ↑, at least twice more; ↓, at least twice less. Case, patient with the indicated antibody positivity; Control, group used as comparator that is negative for that specific antibody, positive for another antibody when indicated in brackets. NA, not assessed.

#### 3.3.1 Anti-PM/Scl

Anti-PM/Scl antibodies are found in 0% to 13% of SSc patients (24, 45, 120, 122–131). A 2017 meta-analysis reported that 31% of scleromyositis patients were anti-PM/Scl positive (132). A similar prevalence was reported in independent cohorts published after 2017 (18, 37, 59).

As compared with anti-PM/Scl antibody-negative SSc patients, patients with anti-PM/Scl antibodies, in addition to having more frequent muscle involvement, were younger at disease onset and more frequently presented with ILD, arthritis, calcinosis and telangiectasias. In contrast, skin involvement was more frequently limited (sclerodactyly or puffy hands), gastrointestinal involvement was less frequent and less severe (91, 120, 122–124, 131, 133, 134), and DM rash was more frequent (91).

Despite a higher incidence of ILD, anti-PM/Scl was associated with a better response to treatment of ILD, and survival was better in this subgroup (91, 133–135).

Even though anti-PM/Scl patients may have histologic evidence of fibrosis, other histopathologic features (e.g. inflammation or necrosis) were prominent (59, 64). Moreover, in the majority of anti-PM/Scl positive patients from the Pittsburgh Scleroderma Databank (58%), inflammatory changes were found on muscle biopsy (120). In accordance with these histopathological findings, myositis had a favorable outcome when treated with CS alone or in combination with azathioprine in most cases (136).

#### 3.3.2 Anti-Ku

The prevalence of anti-Ku antibodies in SSc rangs from 1 to 16% (123, 125, 126, 129–131, 137–146), and from 38% to 55% in scleromyositis patients (41, 142, 147). Several studies demonstrate the association between anti-Ku antibodies and scleromyositis (125, 130, 131, 137, 142, 148–151).

In addition, as compared with anti-Ku antibody-negative SSc patients, SSc patients with anti-Ku antibodies presented more frequently with ILD (137, 138, 140), heart involvement (69, 129), PAH (129, 130, 140), gastrointestinal involvement (140, 149) and arthritis (130, 137). They had more frequent limited skin involvement (123, 130, 137, 140) and less frequent vascular involvement (fingertip ulcers and telangiectasias) (125, 129, 130, 137, 143). Despite more frequent cardiopulmonary involvement, no difference in survival was found between subjects with and without single-specificity anti-Ku antibodies (140).

This autoantibody has been associated with a necrotizing pattern on muscle biopsy (79, 152). Patients with anti-Ku myositis have been reported to have a sustained response to CS in one study (12).

#### 3.3.3 Anti-U1RNP

The prevalence of anti-U1RNP autoantibodies in SSc ranged from 5% to 12% (42, 45, 120, 122, 123, 129–131, 153–155). It was greater in African American SSc patients (29-32%) (126, 155) and in scleromyositis patients (10%-46%) (41, 42, 72).

In addition to muscle involvement, SSc patients with anti-U1RNP antibodies were younger at disease onset (120, 125, 131, 154) and presented more frequently with ILD (153, 156), PAH (123, 125), arthritis (125, 131, 153, 154) and limited skin involvement (120, 154) when compared with anti-U1RNP negative patients.

In accordance with the increased prevalence of ILD and PAH, the survival of patients with anti-U1RNP is reduced as compared with SSc patients with anti-centromere antibodies (123). However, in PAH-SSc patients, anti-U1RNP positivity was associated with better functional outcomes along with better 5-year and 10-year survival rates. On multivariate analysis including age at PAH diagnosis, sex, World Health Organization functional class, forced vital capacity % predicted value and hemodynamic parameters, anti-U1RNP positivity remained negatively associated with mortality in the subgroup of PAH-SSc patients (157).

Similarly to anti-Ku, this autoantibody has been associated with a necrotizing pattern on muscle biopsy (79). Patients with anti-U1RNP myositis have been reported to have a sustained response to CS in one study (12).

#### 3.3.4 Anti-U3RNP (fibrillarin)

The prevalence of anti-U3RNP (fibrillarin) antibodies in SSc ranged from 1% to 14% (120, 125, 131, 154, 158–163) and is greater in African American patients (17-45%) (126, 155, 161).

In scleromyositis anti-U3RNP antibodies had a prevalence of 6% (72).

In addition to muscle involvement, as compared with anti-U3RNP-negative SSc patients, patients with anti-U3RNP antibodies were more frequently male (158, 159, 164) and younger at disease onset (120, 131, 158, 161, 164). They more frequently presented with diffuse cutaneous disease (125, 131, 163), digital ulcers, calcinosis (161), severe gastro-intestinal involvement (159, 161, 163, 165), myopericarditis and PAH (120, 158, 161, 165), which was the most common cause of death (158).

Some studies found a positive association with ILD (120, 159, 163) as well as with arthritis (120, 163), but others a negative association (125, 164, 165) with them.

This autoantibody has been associated with fibrosis on muscle biopsy (72). This pattern has been reported to be less responsive to immunosuppressive treatment such as CS, methotrexate or mycophenolate (72).

#### 3.3.5 Anti-RuvBL1/2

Anti-RuvBL1/2 are rare SSc-related antibodies (1%-2%) (149). Only 51 such patients have been described (166) and about 60% of them were diagnosed with scleromyositis (149, 166, 167). In addition to muscle involvement, these patients were characterized by older age at disease onset, male sex and diffuse skin thickening. Cardiac, gastrointestinal and peripheral vasculature involvements we also described.

#### 3.3.6 Anti-SMN

Recently, a novel autoantibody targeting survival of motor neuron complex (anti-SMN) has been described in a few scleromyositis patients (167–169).

Scleromyositis patients with anti-SMN autoantibodies displayed proximal weakness, elevated serum CK levels and abnormal EMG (100%), arthritis (60%), SSc calcinosis (60%) and lSSc (80%) (167). In these patients, a nuclear dots pattern in indirect immunofluorescence (AC6/7 according to the International Consensus on Antinuclear Antibody standardized nomenclature) (170) was a useful screening test for identifying anti-SMN autoantibodies (167).

Additionally, anti-SMN autoantibodies have recently been reported in up to 59% of patients in an anti-U1RNP-positive mixed connective tissue disease cohort (171, 172). Interestingly, the presence of anti-SMN autoantibodies, especially with high titers, was associated with a higher prevalence of scleromyositis compared to patients without anti-SMN autoantibodies (171).

#### 3.3.7 Seronegative scleromyositis

Almost 50% of scleromyositis patients do not have SS-associated autoantibodies (21, 28). This subgroup has been called “seronegative” and represents a that is difficult to recognize by clinicians as typically SSc skin involvement is frequently lacking (167). Cancer (20%) and deaths (15%) were common (21), highlighting the importance of correct diagnosis and adequate treatment. Furthermore, these “seronegative” patients provide the opportunity to discover new autoantibodies that could improve the accuracy of diagnosis and potentially shed light on unknown pathogenetic aspects of scleromyositis.

### 3.4 Treatments

Randomized controlled trials of cyclophosphamide, mycophenolate mofetil, tocilizumab, rituximab, nintedanib, as well as hematopoietic stem cell transplantation (HSCT), have demonstrated efficacy (stabilization or improvement) on skin thickening and/or ILD in SSc patients (173). In contrast, the level of evidence on the effect of these treatments for muscle involvement in SSc is very low, based on uncontrolled case series and data derived from other AIM subtypes. Currently, no consensual clinical practice guidelines are available for scleromyositis management (174, 175).

#### 3.4.1 Corticosteroids

High dosage CS are generally used to treat AIM patients (174). In the setting of scleromyositis, the benefits of CS should be balanced with the potential side effects as CS have been associated with an increased risk of SRC in SSc (176, 177). In a 2012 systematic review of the available literature, the rate of SRC in SSc treated with CS was approximately twice the expected rate for SSc in general (2% vs 0.94%) (177). Moreover, in the International Scleroderma Renal Crisis Survey, for every milligram of prednisone, the risk of death increased by 4% (176). Whether this is causal or the result of confounding with underlying disease severity remains unresolved (103). On the other hand, CS were effective in 78 to 100% of scleromyositis patients (12, 27, 32, 34). The EULAR experts recognized that CS are part of the therapeutic strategy in the management of musculoskeletal involvement, although the evidence regarding their efficacy in SSc is limited. They recommend that patients with SSc treated with corticosteroids should be carefully monitored with respect to the development of SRC (178). In scleromyositis, sero-pathological features may help to predict CS response of myositis. In particular, necrosis and inflammation shown on muscle biopsy were associated with a response to CS in more than 90% of cases whereas the absence of these lesions, also described as “fibrosing myopathy” (72), is associated with an improvement in only one third of cases (27). In accordance with their association with necrosis and/or inflammation on muscle biopsy (79, 152), anti-PM/SCL, -U1-RNP and -Ku autoantibodies were associated with sustained response of the myositis to CS (12). On the other hand, possibly because of their association with fibrosis on muscle biopsy (72), anti-Scl70 and anti-U3-RNP were associated with a poor response of the myositis to CS (72).

#### 3.4.2 Immunomodulatory drugs

Consistent with its efficacy in SSc-ILD (179), the most frequently used immunomodulatory drug in recent series of scleromyositis patients was mycophenolate mofetil (72). However, no data have demonstrated the efficacy or the corticosteroid-sparing effect of this drug in SSc muscle involvement, nor in other AIM subgroups. Methotrexate and azathioprine were also used (27, 72, 121). Efficacy and corticosteroid-sparing effect have been only demonstrated for methotrexate in the setting of juvenile dermatomyositis (180).

#### 3.4.3 Intravenous immunoglobulins

Intravenous immunoglobulins (IVIg) have demonstrated efficacy in refractory DM patients (181, 182) and uncontrolled data indicated some efficacy in other subtypes of refractory myopathies (183). In a series of 52 patients with scleromyositis, 18 patients (34.6%) who received IVIg were taking lower CS doses at one year and at the end of follow-up than patients who did not receive IVIG, despite higher maximal CS dose ever, indicating a CS-sparing effect (184).

#### 3.4.4 Rituximab

In a randomized controlled trial of refractory AIM, rituximab given early failed to demonstrate any superiority versus rituximab given 2 months. However, 83% of all the patients improved during the trial after a median interval of 20 weeks (185). Rituximab has been included as a therapeutic option in an expert consensus for several refractory AIM subgroups (183, 186). Rituximab has recently demonstrated efficacy for skin and ILD treatment in SSc (187). This treatment was shown to be effective in SSc patients with muscle involvement in case series (188).

#### 3.4.5 Abatacept

A randomized control trial failed to demonstrate efficacy of abatacept on skin thickening in patients with SSc (189), although recent analysis by intrinsic gene expression signatures seemed to show an effect in patients in the inflammatory subset (190). Preliminary results of a recent randomized control trial indicate efficacy of abatacept in a subgroup of patients with IMNM (191). In a case series of 7 scleromyositis patients with refractory muscle involvement treated with abatacept, after a follow-up of 18 months, the myopathy tended to improve as judged on the myopathy disease activity assessed by both patients (57/100 versus 19/100) and the physicians (28/100 vs 12/100), as well as on the median serum levels of CK (456 U/L, range 166–1800 versus 192, range 109–402). Yet, the differences were not statistically significant and muscle strength was unchanged (121).

#### 3.4.6 Autologous hematopoietic stem cell transplantation

Among patients with early dSSc, autologous HSCT has been associated with increased treatment-related mortality in the first year as compared to cyclophosphamide, but with significant long-term event-free survival benefit (192, 193). Efficacy on muscle involvement was not assessed. One patient developed myositis in the HSCT group versus none in the cyclophosphamide group (192). It is currently unknown whether HSCT may be a therapeutic option in fibrosing myopathy, a condition in which other treatments have been reported to fail in two thirds of the cases (27, 72).

## 4 Discussion

We reviewed in-depth studies reporting muscle involvement in SSc.

**Figure 2:**
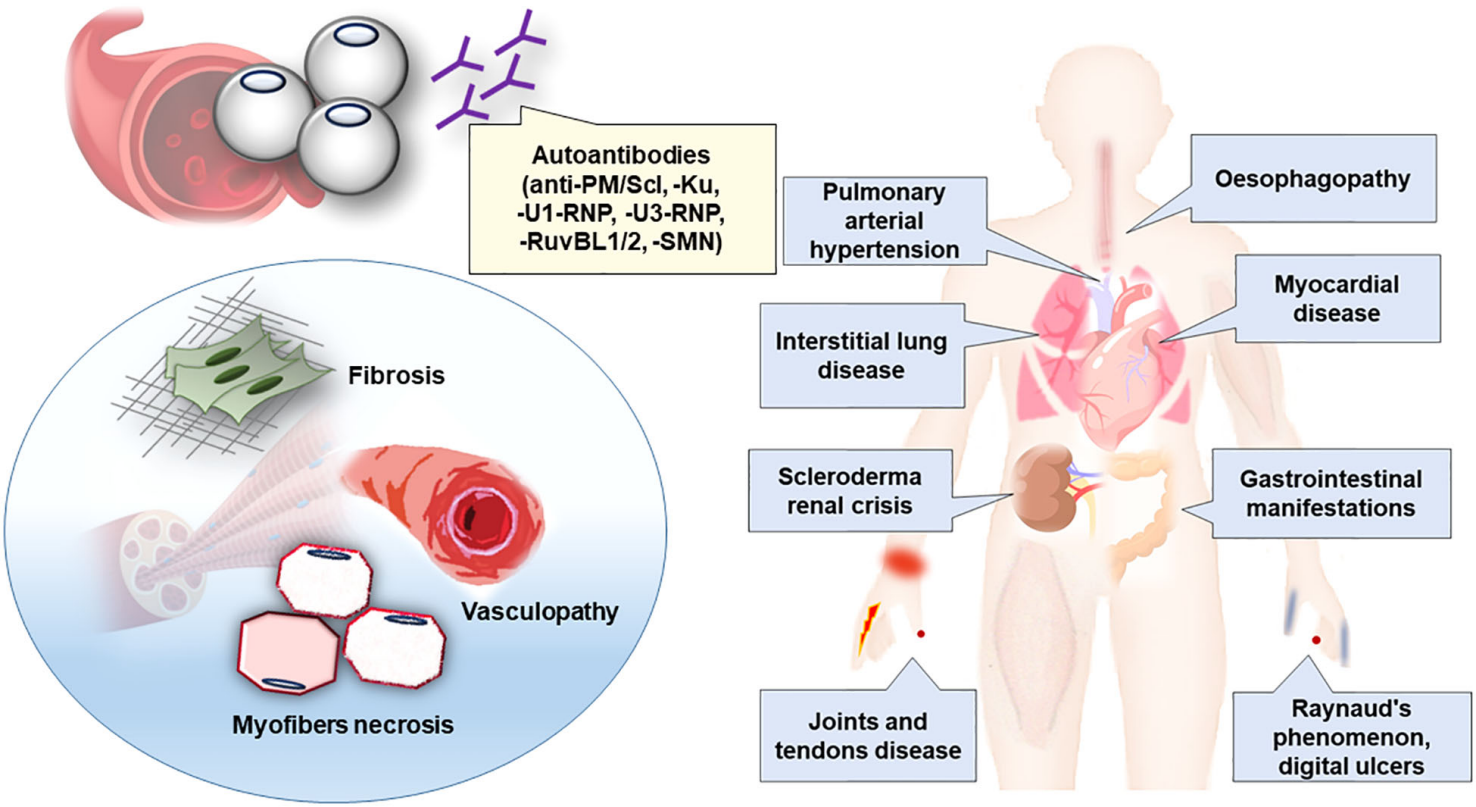
Clinico-sero-pathological definition of scleromyositis. Clinical phenotype, autoantibodies and main histopathological lesions associated to scleromyositis have been summarized.

Although the definition of scleromyositis in the literature is heterogeneous, our review provided evidence that an additional disease subgroup should be recognized within both the SSc and AIM spectrum that has been termed “scleromyositis” (20, 21, 63, 167). Specifically, recent data indicate that the hallmarks of scleromyositis extend far beyond an overlap of AIM and SSc. Additionally these data include characteristic extra-muscular clinical, serological and histopathological features (17, 18, 20, 21, 24–28, 63, 72, 82, 149, 167) supporting scleromyositis as a distinct entity having important implications for prognosis and management of patients (27, 29, 72, 82, 104) (**Table 5**; **Figure 2**).

**Table 5:** Distinguishing clinical, serological and pathological features of scleromyositis.

DOMAIN	FEATURES
Clinical	Muscle involvement and its features Weakness: generally symmetrical and proximal both in the upper and lower limbs (reported upper limbs weaker than lower); axial (head drop syndrome and/or camptocormia in up to one third of patients); more rarely distal CK serum levels: increased (CK levels > 5 times the ULN associated with better response to CS) ENMG: generally myopathic pattern in proximal than in distal muscles (spontaneous activity associated with highest CK levels); neuropathic pattern in up to 30% of patients Most common extra-muscular involvement and their features Skin: generally diffuse cutaneous scleroderma (but limited or even sine scleroderma is possible) Respiratory : interstitial lung disease, respiratory muscles Heart: myocarditis Blood vessels: pulmonary arterial hypertension Gastro-intestinal: severe manifestations Joints and tendons: tendon friction rubs and synovitis
Serological	Positively associated autoantibodies Anti-PM/Scl, anti-Ku, anti-U1RNP, anti-U3RNP, anti-RuvBL1/2, anti-SMN Negatively associated autoantibodies Anti-CENP-B Seronegative Up to 50% of patients
Muscle pathology	Specific lesions Vasculopathy: prominent basement membrane reduplication (≥4 layers in >50% of capillaries) at electron microscopy Prognostic lesions Muscle fiber necrosis (associated with good response to treatment), fibrosis (associated with refractoriness to treatment)

CK, creatine-kinase; CS, corticosteroids; ENMG, electroneuromyography; ULN, upper limit of normal.

Similarities and differences between scleromyositis and other AIM such as anti-SRP/-HMGCR IMNM and ASS must be emphasized in order not to confuse these conditions.

Although IMNM, as defined at the pathological level by the 2005 ENMC criteria (78), was the most common finding in muscle biopsy of scleromyositis patients, important differences were identified at the serological level by the more recent 2016 ENMC criteria (183). Muscle fiber necrosis has been associated in scleromyositis with anti-Ku, anti-PM/Scl and anti-U1-RNP autoantibodies whereas in IMNM fiber necrosis has been associated with anti-SRP and anti-HMGCR autoantibodies. Importantly, muscle biopsies of scleromyositis patients additionally display vasculopathy and fibrosis that are not features of anti-SRP and -HMGCR IMNM. Finally, extra-muscular features are frequent in scleromyositis, are linked to poor prognosis and require careful management while such features are exceptional in anti-SRP and anti-HMGCR IMNM patients. Interestingly, the two conditions are distinct even at the genetic level. Thus, anti-PM/Scl positive AIM has been linked with HLA-DQB1*02 (194). By contrast, anti-HMGCR -IMNM has been associated with HLA-DRB1*11 allele (194, 195) and no significant association with classical HLA alleles has been found for anti-SRP-IMNM.

Scleromyositis and ASS share some overlap clinical features, including ILD, arthritis, and Raynaud’s phenomenon (6, 12). Yet, the other clinical (i.e. mechanic’s hands and constitutional symptoms in ASS versus skin thickening, digital ulcers, telangiectasias, calcinosis, myocarditis and PAH in scleromyositis), serological (autoantibodies targeting aminoacyl-tRNA synthetases in ASS versus SSc-associated autoantibodies, as detailed above, in scleromyositis) and histopathological (perifascicular fibers necrosis in ASS versus necrotizing myopathy and vasculopathy in scleromyositis) (6, 20, 196) characteristics indicate that ASS and scleromyositis are two distinct entities. Consistently, at the genetic level, the HLA-B*0801 and the HLA-DRB1 03:01 alleles are associated with anti-Jo-1 ASS, while anti-PM/Scl scleromyositis is associated with HLA-DQB1 02:01 allele (194, 197).

Finally, overlaps between AIM and autoimmune diseases other than SSc (especially SLE and SjS) have also been reported (61, 198–204). Compared to scleromyositis, these associations are rarer. Yet, some clinical, histopathological, serological characteristics have been highlighted that have also implication for management and prognosis.

In patients with SLE, a biopsy proven myositis according to Bohan and Peter criteria (52, 53) has been found in only 3% of cases (201, 205). In contrast with scleromyositis patients, the frequency of ILD is lower in patients with SLE/myositis. On the other hand, the frequency of glomerulonephritis is higher (up to 39%), thus representing a hallmark life-threatening complication of this group (54, 200, 206). Anti-U1RNP are the most common antibody associated with SLE/myositis overlap syndrome (200, 201, 205, 207). Some SLE/myositis patients have anti-Ku antibodies (137, 150, 151, 200). In contrast with anti-U1RNP and anti-Ku scleromyositis patients, anti-U1RNP and anti-Ku SLE/myositis patients generally test positive for anti-dsDNA antibodies. This associations helps to predict whether lung (scleromyositis) or kidney (SLE/myositis) will drive prognosis (151, 199, 200, 206).

In patients with primary SjS, AIM is frequently suspected (10%), but rarely confirmed (1%) (61). More frequently, SjS is found in AIM (34% of patients) (198). When confirmed, AIM in SjS patients is more likely to be IBM whose diagnosis is fundamental since conventional immunomodulating agents are not effective and may even increase the risk of progression toward disability (208), while IBM-specific treatments may slowdown the progression of that disease (209). This association has been demonstrated both in patients with primary SjS (0.5% vs. 2.01/100 000 [95% CI 1.51-2.69] in the general population) (210) and in patients with AIM and secondary SjS (24% vs 6% in AIM patients without SjS) (61, 198, 211). Anti-cN1A is frequent in both SjS and IBM (212). Yet, in AIM patients, the association between anti-cN1A and SjS is independent of IBM raising caution about using anti-cN1A for the diagnosis of IBM in SjS patients (198, 211).

A systematic review was not performed because there is currently no consensual definition of scleromyositis. Thus, inclusion and exclusion criteria of studies selection were not strictly defined for the purpose of comprehensiveness. The literature research was performed without any restrictions in terms of publication period, type of study, nor setting. The key words were chosen to cover the entire scope of SSc and AIM. Our review revealed that SSc and AIM were classified using heterogeneous criteria, that probably accounts of the wide ranges in the reported prevalences.

In light of this review, **Table 5** is proposed highlighting the distinguishing clinical, serological and pathological features of scleromyositis thus far.

## 5 Conclusion

Scleromyositis is an emerging entity in the SSc and AIM spectrum that is important for clinicians to recognize because it has many potential organ involvements (including lung, heart, gastrointestinal, skin and joint) and requires a tailored management different from AIM or SSc alone, especially regarding the risk of SRC upon CS exposure. Several autoantibodies and muscle histopathological findings are hallmarks of scleromyositis and, moreover, they assist in the prediction of extramuscular outcomes and response to treatment. An integrated clinico-sero-pathological approach is proposed (**Figure 2**) to recognize this novel subgroup.

## Author contributions

MG: Data curation, Formal analysis, Investigation and Writing - original draft, Conceptualization, Methodology, Project administration, Supervision and Validation, Writing - review and editing. BE Conceptualization, Methodology, Writing - review and editing. VL: Methodology, Writing - review and editing. FL: Data curation, Formal analysis. YT: Writing - review and editing. MH: Conceptualization, Methodology, Supervision and Validation, Writing - review and editing. JL-S: Conceptualization, Methodology, Supervision and Validation, Writing - review and editing. BG: Writing - original draft, Project administration, Supervision and Validation, Writing - review and editing. OL-C: Conceptualization, Methodology, Supervision and Validation, Writing - review and editing. AM: Data curation, Formal analysis, Investigation and Writing - original draft, Conceptualization, Methodology, Project administration, Supervision and Validation, Writing - review and editing. All authors contributed to the article and approved the submitted version.
